# Inhibition of Lipid Peroxidation of Kiwicha (*Amaranthus caudatus*) Hydrolyzed Protein Using Zebrafish Larvae and Embryos

**DOI:** 10.3390/plants7030069

**Published:** 2018-09-02

**Authors:** Rubén Vilcacundo, Daniel Alejandro Barrio, Lucrecia Piñuel, Patricia Boeri, Andrea Tombari, Adelita Pinto, James Welbaum, Blanca Hernández-Ledesma, Wilman Carrillo

**Affiliations:** 1 Laboratory of Functional Foods, Faculty of Foods Sciences and Engineering, Technical University of Ambato, Av. Los Chasquis y Rio Payamino, Campus Huachi, Ambato 1801334, Ecuador; rd.vilcacundo@uta.edu.ec; 2 CIT-RIO NEGRO Sede Atlántica, Universidad Nacional de Rio Negro (UNRN-CONICET), Don Bosco y Leloir s/n CP 8500, Rio Negro Viedma, Argentina; drbarrio@unrn.edu.ar (D.A.B.); lpinuel@unrn.edu.ar (L.P.); pboeri@unrn.edu.ar (P.B.); atombari@unrn.edu.ar (A.T.); 3 Department of Research, Faculty of Health Sciences, Technical University of Babahoyo, Av. Universitaria Km 21/2 Av. Montalvo., Babahoyo 120301, Ecuador; apinto@utb.edu.ec; 4 Department of Chemistry and Biochemistry, Texas Tech University, Lubbock, TX 79413, USA; james.welbaum@ttu.edu; 5 Research Institute of Food Science (CIAL-CSIC-UAM), Nicolás Cabrera 9, 28049 Madrid, Spain; b.hernandez@csic.es

**Keywords:** kiwicha, simulated gastrointestinal digestion, protein concentrate, lipid peroxidation, zebrafish embryos

## Abstract

Amaranth protein concentrate (APC) was hydrolyzed under in vitro gastrointestinal conditions. APC proteins were partially degraded by pepsin at pHs 1.2, 2.0, and 3.2. During the intestinal phase (pepsin/pancreatin enzymes at pH 7.0), no polypeptide bands were observed in the gel, suggesting the susceptibility of amaranth proteins to the action of digestive enzymes. The potent in vitro inhibition of lipid peroxidation, shown by the gastric and intestinal digests, was confirmed in the zebrafish larvae, with a 72.86% reduction in oxidation of lipids in the presence of the gastric hydrolysate at pH 2.0, compared to a 95.72% reduction in the presence of the gastrointestinal digest. APC digests were capable of reducing reactive oxygen species (ROS) production in the zebrafish embryo model with a value of fluorescence of 52.5% for the gastric hydrolysate, and 48.4% for the intestinal hydrolysate.

## 1. Introduction

Kiwicha (*Amaranthus caudatus*) is a pseudocereal from the Andean region used by pre-Columbian cultures for centuries. Similarly, to other Amaranth species, this crop is currently gaining popularity due to its genetic variability, agronomic advantages, and excellent nutritional properties, such as a high percentage of protein (14–18%, *w*/*w*) with high digestibility, and an excellent balance of amino acids [1].

Peptides are protein fragments that are released during gastrointestinal digestion and food processing. These peptides can exert different biological properties such as antioxidant, anti-inflammatory, anti-hypertensive, and antibacterial activities. In addition to their nutritional value, amaranth proteins have been recently recognized as a source of bioactive peptides.

Several peptides have been identified after their release from *A. hypochondriacus* and *A. mantegazzianus* proteins, and characterized for their angiotensin-converting enzyme inhibitory, anti-inflammatory, and antioxidant activities [2,3,4]. Two antibacterial peptides named Ac-AMP1 and Ac-AMP2 were isolated from seeds of *A. caudatus*, demonstrating activity against *Bacillus megaterium* ATCC 13632, *Escherichia coli* strain HBl01, *Erwinia carotouora* strain 3912, and *Sarcina lutea* ATCC 9342. These peptides also presented antifungal activity against *Alternaria brassicola* MUCL 20297, *Ascochyta pisi* MUCL 30164, *Botrytis cinerea* MUCL 30158, *Colletotrichum lindemuthianum* MUCL 9577, *Fusarium culmorum* IMI 180420, *Trichoderma hamatum* MUCL 29736, and *Verticillium dahliae* MUCL 19210 [5].

Large quantities of reactive oxygen species (ROS), such as O_2_^•−^, HO_2_^•^, and ^•^OH, induce damage to DNA and cellular lipid oxidation, resulting in oxidative stress. That stress is implicated in degenerative diseases such as Alzheimer’s and Parkinson’s diseases [6,7,8]. Butylated hydroxy anisole (BHA), butylated hydroxytoluene (BHT), tertiary butylhydroquinone (TBHQ), and propyl gallate (PG) are antioxidant chemical substances that are widely used in the pharmaceutic and food industry to prevent lipid oxidation. In the last decade, their high cytotoxicity demonstrated in different in vivo models have restricted their use in some countries [9,10,11]. Currently, there is an interest in the search for natural substances with antioxidant capacities to be used in the food and pharmaceutical industry.

Lipid oxidation is named as lipid peroxidation with the oxidation of unsaturated fatty acids of the cell membranes. In different studies, many methods to measure lipid peroxidation have been described. These methods are used to directly measure the antioxidant capacity of compounds (β-carotene bleaching assay, iodometric hydroperoxide measurement, thiobarbituric acid-reactive substances (TBARS) assay, ultraviolet spectroscopic measurement of conjugated dienes methods) [12,13,14]. The TBARS assay is a common spectrophotometric method used to measure lipid peroxidation, due to its easy methodology and low cost [15,16]. Different studies have described the antioxidant activity and lipid peroxidation inhibitory capacity of isolated proteins from animal and vegetable sources, such as quinoa, rice, fish, egg, milk proteins, *A. hypocondriacus*, and *A. mantegazzianus* and soybean [17,18,19,20,21]. Zebrafish is an animal model that is used for different purposes in medicine, biotechnology, pharmacy, and toxicology. Zebrafish eggs, larvae, and embryos present morphological characteristics such as fast growth, transparent bodies, low maintenance costs, and similarity to human genetic expression, that facilitate their use. In past years, the zebrafish model has been used to evaluate animal development and to replicate human degenerative diseases. The zebrafish model has also been used to evaluate the reduction of ROS [22,23,24]. It has been described that protein concentrate hydrolysates and peptides can reduce lipid peroxidation using the zebrafish larvae and embryos models [25]. Peptide sequences identified in lysozyme protein and gastrointestinal digests from quinoa (*Chenopodium quinoa* Willd) protein showed inhibition of lipid peroxidation using the zebrafish larvae model [26].

The aim of this study was to evaluate simulated gastrointestinal digestion of *A. caudatus* and its potential to release peptides with a capacity to reduce lipid peroxidation and ROS, using the zebrafish model.

## 2. Materials and Methods

### 2.1. Materials

Pepsin (4500 U/mg), pancreatin (10,000 U/mg), 1,3,3-tetraethoxypropane (malonaldehyde, MDA), thiobarbituric acid (TBA), dimethyl sulfoxide (DMSO), 2,2’-azobis (methylpropionamide)-dihidrochloride (AAPH), BHT, and 2,7-dichlorofluorescein diacetate (DCFH-DA) were obtained from Sigma-Aldrich (St. Louis, MO, USA). The other reactants used in this study were of analytical grade.

### 2.2. Amaranth Protein Concentrate (APC) Preparation

APC was prepared following the protocol of Acosta et al. [27]. Kiwicha flour (Gramolino, Quito, Ecuador) was dissolved in distilled water (1:10, *w*/*v*) at pH (8.0). The mix was shaken for 1 h, and centrifuged at 4500× *g* (30 min, 25°C). The supernatant was separated, its pH was adjusted to pH 4.0, and it was centrifuged at 4500× *g* for 20 min at 4 °C. The pH of the retentate was neutralized with NaOH 0.1 M, lyophilized, and stored at −80 °C until further analysis. APC protein content was determined using the bicinchoninic acid (BCA) method.

### 2.3. In Vitro APC Gastrointestinal Hydrolysis

APC (5 mg/mL) was subjected to an in vitro gastrointestinal hydrolysis that simulated physiological conditions. Firstly, the APC sample was dissolved in simulated gastric fluid (SGF, 0.35 M NaCl) at pH 1.2, pH 2.0, and pH 3.2, containing pepsin at an enzyme:substrate ratio of 2000 U/mL, then incubated for 120 min at 37 °C. The reaction was stopped by heating (80 °C, 5 min). The gastric hydrolysate obtained at pH 3.2 was used for the intestinal phase. One milliliter of gastric hydrolysate was combined with one milliliter of pancreatin solution (100 U/mL) containing bile salt and sodium chloride. The mix was incubated at 37 °C for 2 h. The reaction was stopped using the heat treatment (80 °C for 5 min) [28].

### 2.4. APC and Hydrolysates Analysis Using SDS-PAGE Electrophoresis and RP-UHPLC Chromatographic Methods

SDS-PAGE electrophoresis of APC and its hydrolysates were analyzed using a Mini-Protean Cell electrophoresis instrument (Bio-Rad, Hercules, CA, USA) with 12% polyacrylamide gels. The gels were stained with Coomassie Brilliant Blue G-250 overnight with agitation. The molecular weight of protein present in APC and the hydrolysates were determined using a marker that contained protein standards with molecular weights from 6.5–200 kDa [29]. The samples were analyzed by RP-UHPLC using an Agilent 1200 infinity UHPLC system (Agilent Technologies, Waldbron, Germany). The column used was Zorbax EC C18 (Agilent Poroshell 120, 4.6 × 50 mm × 2.7 µm). All samples were eluted at a flow of 1.0 mL/min with a lineal gradient of solvent B (acetonitrile) in the solvent A (milli-Q water) from 0–70% in 12 min. A PDA detector was used to analyze the samples (wavelength of 220 nm and 280 nm). All samples were centrifuged at 10,000 rpm for 2 min at 4 °C and the soluble fraction was filtered and analyzed by UHPLC [30].

### 2.5. Evaluation of In Vitro Lipid Peroxidation of APC and Its Hydrolysates

Lipid peroxidation inhibition of APC and its gastrointestinal hydrolysates was evaluated with the in vitro TBARS method. 500 mg of sacha inchi oil was oxidized by heating at 65 °C for one week. APC and digests (2.5 mg/mL), the negative control sacha inchi (*Plukenetia volubilis*) oil without sample, and a positive control BHT, (0.1 mg/mL) were used in this study. One milliliter of sample was mixed with 1.0 mL of 1% TBA solution (TBA was dissolved in distilled water with 5% acetic acid). The solution of sample/TBA was heated at 95 °C for 1 h. The absorbance of the mixture was measured at 532 nm using a spectrophotometer (Thermo Scientific Evolution 200, Madison, WI, USA) [31]. Sacha inchi oil was analyzed by GC-MS and its fatty acid composition was 3.98% palmitic acid, 3.12% stearic acid, 8.58% oleic acid, 34.98% linoleic acid, and 47.04% linolenic acid.

### 2.6. Evaluation of In Vivo Lipid Peroxidation Inhibition in Zebrafish Larvae in the Presence of H_2_O_2_ and Ethanol of APC and Hydrolysates

The zebrafish colony wild type was established in the laboratory of the Technical University of Ambato (Ambato, Ecuador), in a glass aquarium, and provided with a system of filtration and aerator activated carbon for water oxygenation. Adult zebrafish were kept under cycles of 16 h light and 8 h dark. Adult zebrafish were fed with commercial food and the larvae of *Artemia saline.* Zebrafish embryos were obtained by photo-induced spawning over green plants, and were incubated at 28 °C in a fish tank. Early larvae post-fertilization zebrafish of five days were maintained at 28 °C and observed by microscopy.

The lipid peroxidation reduction by APC and its hydrolysates were evaluated by the TBARS method in the zebrafish larvae model as previously described by Carrillo et al. [31]. Zebrafish larvae were put in plastic plates (24-well) using 30 zebrafish larvae/well with APC, gastric, and gastrointestinal hydrolysates (2.5 mg/mL). BHT (0.1 mg/mL) was used as positive control. Groups of 30 zebrafish larvae/well without sample were used as basal control. Lipid peroxidation was commenced by adding 1.0 mL of 500 µM H_2_O_2_ solution or 1.5% ethanol solution. All groups were incubated at 28 °C for 8 h. Then, the medium was changed to remove H_2_O_2_ and ethanol solutions, and 500 µL of Tween 0.1% were added to the wells [32,33]. Zebrafish larvae were mixed and homogenized. A volume of 1.0 mL of 1% TBA was added and the solution was heated at 95 °C for 60 min. The solution was centrifuged at 12,000× *g* for 10 min. The absorbance of the solution was measured at 532 nm. Concentration of TBARS was determined by a standard curve prepared with MDA. The equation obtained was y = 0.0304x + 0.0048 (R^2^ = 0.9964).

The values of TBARS inhibition were calculated as the percentage of reduction of lipid peroxidation in zebrafish larvae homogenate as follows:

% Inhibition of TBARS = [1 − (Ac − As)/Ac × 100],

where Ac = absorbance of basal control and As = absorbance of the sample.

### 2.7. Exposure of Embryos of Zebrafish to APC Hydrolysates and AAPH

Zebrafish embryos of 7–9 h post-fertilization (7–9 hpf) were used. These embryos (group = 4 embryos) were transferred to the wells of a 12-well plate with an osmotic embryo medium E2 1× (15 mM NaCl, 0.5 mM KCl, 1.0 mM CaCl_2_. 2H_2_O, 50 µM Na_2_HPO_4_, 150 µM KH_2_PO_4_, 10 mM MgSO_4_·7H_2_O, 0.7 mM NaHCO_3_, and 0.5 mg/L of methylene blue dissolved in distilled water containing 1.0 mL of vehicle (0.1% DMSO) and 2.0 mg/mL APC hydrolysate for 2 h). Then, zebrafish embryos were treated with 25 mM (AAPH) or AAPH/APC hydrolysate for 24 h post-fertilization (24 hpf) [34].

### 2.8. Estimation of Intracellular ROS Generation and Image Analysis

Generation of ROS in zebrafish embryos without chorion was analyzed using a fluorescent assay (DCFH-DA). DCFH-DA is modified to become DCFH in the presence of cellular peroxidases and is then converted to a highly fluorescent substance (DCF) [35]. Before the assay, the zebrafish embryos were treated in a medium with 0.1% DMSO to permeabilize the chorion of zebrafish eggs. Zebrafish embryos (3–4 hpf) were treated with 2.0 mg/mL gastric and gastrointestinal hydrolysates. 25 mM AAPH was added to the plate that was incubated for 2 h at 28 °C. After incubation with 25 mM AAPH for 2 h, the zebrafish embryo medium was changed, and the embryos developed up to 1 dpf. The zebrafish embryos were transferred to 96 well plates, treated with a DCFH-DA solution (2.0 µL/mL), and the plates were incubated for 2 h in the dark at 28 °C. After incubation, the zebrafish embryos were rinsed in a fresh embryo medium and the chorion membrane was removed with the help of tweezers before visualization. The image of stained embryos was observed using a fluorescent microscope (Leica DM1000 LED, Wetzlar, Germany), with a Moticam 2000 (Taiwan, China) color camera.

### 2.9. Statistical Analysis

Experimental results were presented as means ± standard deviation (*n* = 3). Differences between groups values were determined using one-way ANOVA analysis, followed by the Tukey’s test. All tests were considered with statistical differences at *p* < 0.05 using the software GraphPad Prism 4.

## 3. Results and Discussion

### 3.1. Analysis of APC by SDS-PAGE and RP-UHPLC Methods

**Figure 1:**
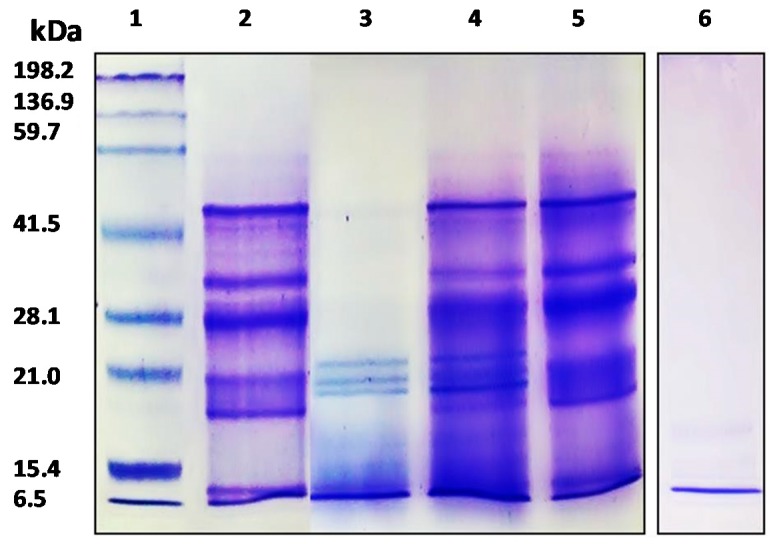
SDS-PAGE electrophoresis analysis of APC and its gastrointestinal hydrolysates. 1: molecular weight standard; 2: APC; 3: gastric hydrolysate (pH 1.2); 4: gastric hydrolysate (pH 2.0); 5: gastric hydrolysate (pH 3.2); 6: Gastrointestinal hydrolysate (pepsin pH 3.2 and pancreatin pH 7.0).

Characterization of APC and its gastrointestinal hydrolysates was carried out by SDS-PAGE and the RP-UHPLC method. The protein concentration of APC was 79.85%. As shown in Figure 1, the APC protein profile (lane 2), showed bands with molecular weights between 6500 to 50,000, being very similar to the image obtained by Martínez and Añón (1996) for *A. hypocondriacus* proteins, although the band with a molecular weight of 78,000 kDa corresponding to a subunit from 7S globulin was not observed in our study. The profile shows one band of 10 kDa corresponding to albumin, followed by 11S basic with two bands of 17.3 kDa and 20.8 kDa, two bands corresponding to the 11S acid fraction with a molecular weight of 27.7 kDa and 33.2 kDa, and finally one band of 45.9 kDa corresponding to 7S globulin [36]. Bands with molecular weights between ≈ 45,000 and ≈ 25,000 corresponded to α- and β-peptides of 11S globulin (amarantin) as previously reported by Thanapornpoonpong et al. (2008). The acidic and basic subunits of this protein were visible at 34,000 and 20,000, respectively [37]. Bands with molecular weights lower than 18,000 correspond to albumin components. These albumins were partially resistant to the action of pepsin when kiwicha proteins were incubated with this enzyme at pH 1.2 (lane 3). However, no bands corresponding to globulins were visible, indicating that these proteins were sensitive to the action of this gastric enzyme. At higher pHs (pH 2.0 and pH 3.2), proteins were resistant to gastric digestion (lanes 4 and 5, respectively). After sequential incubation with pepsin at pH 3.2 and pancreatin, no bands were observed in the gel, indicating that during the duodenal phase kiwicha proteins were completely degraded by proteases contained in the pancreatin preparation. This result is in agreement with Orsini Delgado et al., who reported that the decrease of the total bands intensity observed for the gastroduodenal digest from *A. mantegazzianus* protein was due to the release of peptides with molecular weights lower than the detection limit of the gel [38].

**Figure 2:**
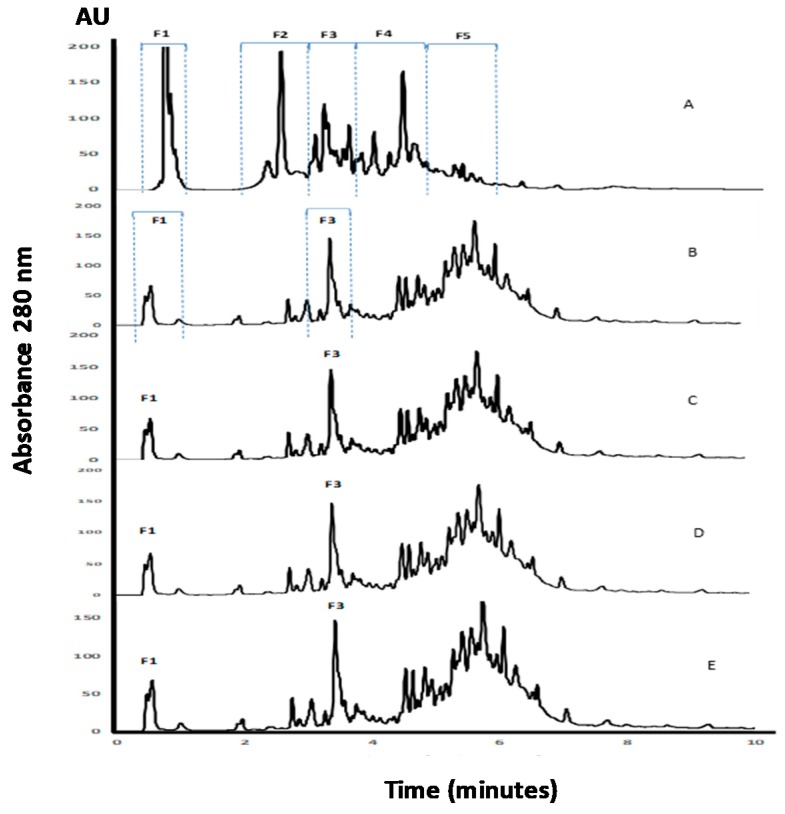
RP-UHPLC analysis of APC and its gastrointestinal hydrolysates. (A) APC, (B) gastric hydrolysate (pH 1.2), (C) gastric hydrolysate (pH 2.0), (D) gastric hydrolysate (pH 3.2), (E) gastrointestinal hydrolysate (pepsin pH 3.2 and pancreatin pH 7.0).

APC, as well as its hydrolysates, were analyzed using the RP-UHPLC method (Figure 2). Fragments contained in the non-hydrolyzed APC eluted during the first 10 min of analysis with five regions of the chromatogram, named as F1, F2, F3, F4, and F5, were clearly observed (Figure 2A). The detection of peaks during the UHPLC analysis of APC before digestion suggests the presence of fragments resulting from the action of endogenous enzymes. The profile changed after incubation with pepsin, with a reduction of the intensities of regions F1 and F2, and a modification of the fragments pattern contained in regions F3–F5 (Figure 2B–D). It is remarkable that the profiles observed for the three gastric hydrolysates were similar, and few changes resulted from the digestion with pancreatin. This could indicate that the fragments released by the action of pepsin under different conditions remained unchanged to a high extent, even after pancreatin addition. Furthermore, the peptides with a higher retention time corresponded to peptides with lower solubility in the organic solvent and/or higher molecular weight, with respect to the initial profile.

### 3.2. Evaluation of Reduction Lipid Peroxidation Using the In Vitro Model

**Figure 3:**
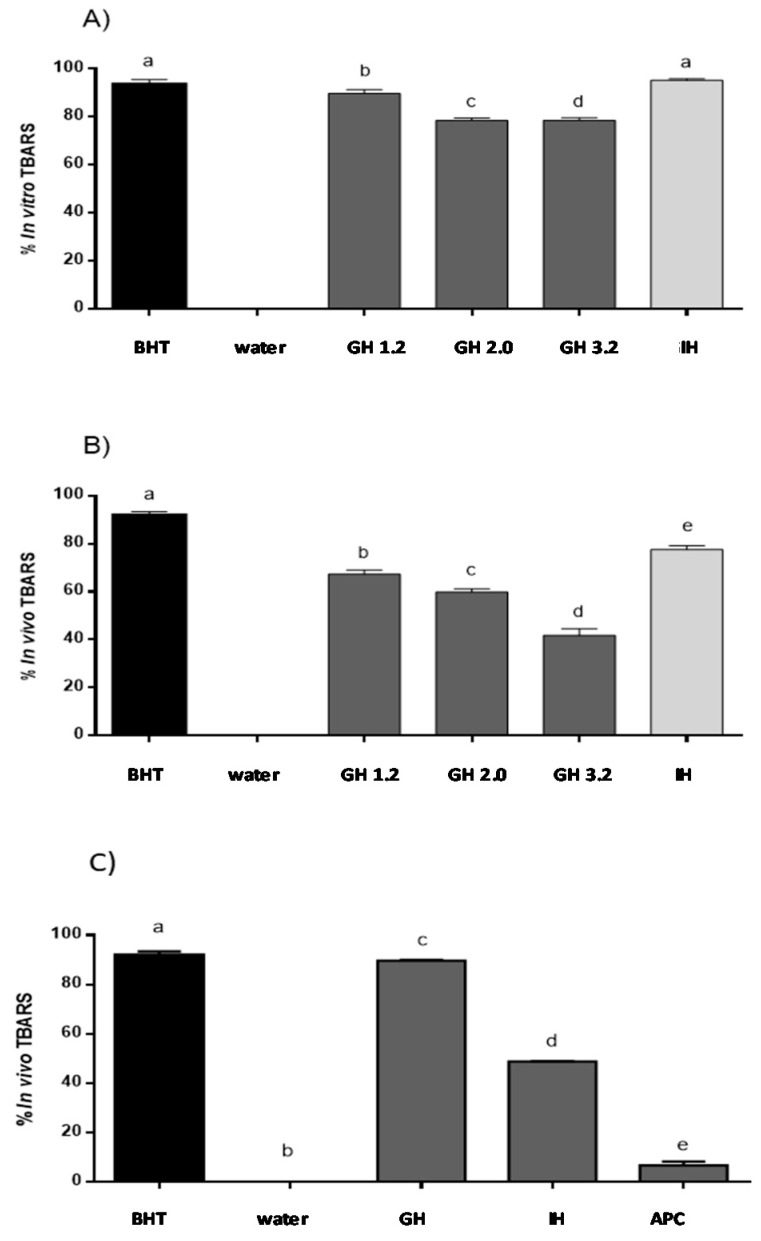
Lipid peroxidation inhibitory capacity of gastrointestinal hydrolysates of APC. (**a**) In vitro TBARS inhibitory capacity (**b**) TBARS inhibitory capacity evaluated in the in vivo zebrafish larvae model with hydrogen peroxide (H_2_O_2_). (**c**) TBARS inhibitory capacity evaluated in the in vivo zebrafish larvae model with ethanol. BHT: positive control, distilled water: negative control, GH (gastric hydrolysate at pH 1.2, pH 2.0 and pH 3.2, IH (gastrointestinal hydrolysate with pepsin pH 3.2 and pancreatin pH 7.0). Data were analyzed using one-way ANOVA and followed by Tukey’s test. Different letters over bars represent statistical differences between groups samples with *p* < 0.05 (*n* = 3).

The lipid peroxidation inhibitory capacity of APC, as well as its gastric and gastrointestinal hydrolysates, was evaluated using the in vitro TBARS method. As shown in Figure 3A, no activity was observed for water used as a negative control, while BHT, used as the positive control, inhibited TBARS up to 93.4% at 0.2 mg/mL. No activity was observed for non-hydrolyzed APC (data not shown). However, after incubation with pepsin at pH 2.0, TBARS was inhibited by 76.86%. Moreover, during the duodenal phase, the antioxidant capacity increased up to 95.72% inhibition, a value that was higher than the one observed for BHT. APC at pH 4.0 presented 7.6% of the inhibition of TBARS in vitro, showing low activity. Recently, the antioxidant activity of peptides released after simulation gastrointestinal digestion of proteins from *A. mantegazzianus* has been described [39]. The authors suggest that the strong increase in the antioxidant activity against free radicals resulting from the action of these peptides could be due to their capacity to inhibit the initiation or propagation of radical reactions.

### 3.3. Evaluation of Inhibition of Lipid Peroxidation Using the In Vivo Zebrafish Larvae Model

Different groups have described that H_2_O_2_ solutions can induce lipid peroxidation using in vitro and in vivo animal models. In this study, H_2_O_2_ was able to cause lipid peroxidation in the zebrafish larvae [23]. The zebrafish larvae model was used as in the in vivo model to confirm the lipid peroxidation inhibition ability of APC hydrolysates (Figure 3B). This ability was evaluated as the protective effect of lipid peroxidation produced using a H_2_O_2_ solution in the zebrafish larvae model. Siddique et al. (2012) reported that lipid peroxidation was induced by H_2_O_2_, and thus, treatments with this chemical at concentration from 1.0 to 200 µM produced an important increase in the production of lipid peroxidation, as compared to the untreated control group [40]. In our study, the inhibition value obtained for BHT was of 90.0%, while distilled water and non-hydrolyzed APC showed no activity. Just as it was observed with the in vitro method, gastric hydrolysate at pH 1.2 showed the highest inhibition capacity, with an inhibition percentage of 67.6%. Gastric hydrolysate at pH 2.0 and pH 3.2 showed inhibition values of 59.8% and 42.3%, respectively. The gastrointestinal hydrolysate produced with pepsin and pancreatin was the most active, inhibiting TBARS up to 77.6%. Low molecular weight peptides released during the two phases of simulated gastrointestinal digestion could be responsible for the highest activity observed. Li et al. (2003) described that 200 µM H_2_O_2_ produced an increase of lipid peroxidation in PC12 cells, and the polysaccharide molecule from *Cordyceps sinensis* was able to revert this increase [41]. Salahudeen et al. (1991) reported that H_2_O_2_ produced lipid peroxidation in a rat whole-kidney homogenate with an increase of TBARS that was then reduced after incubation with pyruvate [42]. Carrillo et al. (2016) described the lipid peroxidation inhibitory capacity of peptides released from lysozyme protein in the same H_2_O_2_-induced zebrafish larvae model. This same effect was observed for native and modified milk protein hydrolysates [25].

The capacity of APC hydrolysates (gastric hydrolysate at pH 2.0 and intestinal hydrolysate at pH 7.0) to inhibit lipid peroxidation was evaluated using the zebrafish larvae model. 1.5% (*v*/*v*) ethanol solution was used to determine the effect in the lipid peroxidation in the zebrafish larvae membranes. Zebrafish larvae 5-day pots-fecundation (dpf) were used to evaluate the APC hydrolysates to inhibit lipid peroxidation with the presence and the absence of ethanol. The MDA content was also determined using a standard curve. BHT inhibited lipid peroxidation by up to 93% in the animal model. APC gastric hydrolysate at pH 2.0 was capable of inhibiting 80.68% TBARS. Intestinal hydrolysate presented a value of 48.86% inhibition of TBARS (Figure 3C). On the other hand, the ethanol solution was capable of inducing the production of lipid peroxidation in zebrafish larvae membranes. TBA solution allowed for the identification of MDA presence, with the formation of a complex of pink colored TBA-MDA. Other aldehydes are produced in lipid peroxidation. These aldehydes could also react with the TBA solution and absorb at 532 nm. For this reason, ROS formation and inhibition was also evaluated using a DCF-DA reaction [43]. Different authors have described that ethanol can produce lipid peroxidation in liver, brain, and different tissues of the body, especially in brain membranes, with these membranes being rich in polyunsaturated acids. Zebrafish larvae present a high brain proportion in the first period of development, when compared to other body tissues. In this study, the polyunsaturated acids from the zebrafish brain can be implicated in the lipid peroxidation observed. It is known that ethanol is a molecule capable of producing lipid peroxidation, with the formation of free radicals in different tissues within the human body [44].

**Figure 4:**
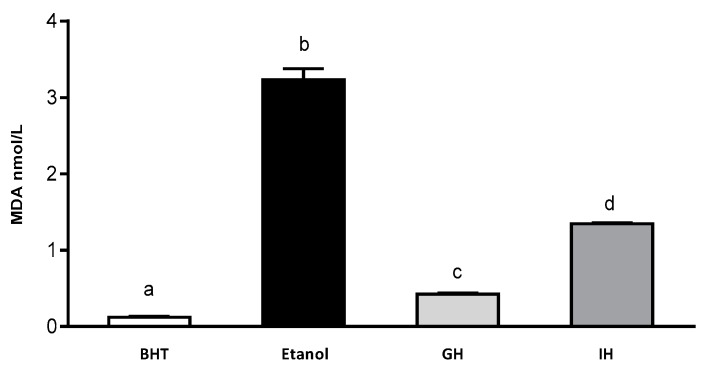
Content of MDA in gastrointestinal hydrolysates from APC. 1.5% ethanol solution, GH (gastric hydrolysate), GIH (gastrointestinal hydrolysate). BHT positive control and ethanol negative control.

Rouach et al. (1987) have described that ethanol is capable of producing oxidative stress by reducing α-thocopherol and ascorbate content. Cytochrome P450 (CYP2E1) is considered to be an inductor of free radicals in brain. Its isoforms are potent generators of free radicals in the brain. CYP2E1 is involved in the mechanism of ethanol-induced liver damage [45,46,47,48,49]. Additionally, the MDA content was determined with the help of a MDA calibration curve (Figure 4). Zebrafish larvae were incubated with 1.5% ethanol (control) and with the presence of 3.2 nmol/L of extract of zebrafish larvae. BHT (positive control) presented 0.1 nmol/L of extract of zebrafish larvae. APC gastric hydrolysate at pH 2.0 had a MDA content of 0.40 nmol/L of extract of zebrafish larvae. The APC gastrointestinal hydrolysate had 1.32 nmol/L zebrafish larvae extract. The APC gastric and gastrointestinal hydrolysates were capable of inhibiting the production of MDA in the zebrafish larvae model.

### 3.4. APC Hydrolysate Protective Effects of Oxidative Stress Produced with AAPH in a Zebrafish Embryo No-Chorion Model

Harman proposed a theory on free radicals, indicating that the increased levels of free radicals with increasing age may cause a progressive accumulation of cellular damage. In normal oxidative phosphorylation, free radicals are produced as by-products. Our brains consume high levels of oxygen, and at the same time produce high levels of free radicals. The presence of ROS indicates oxidative stress in the cells. ROS are associated with different age-associated diseases. It is known that a healthy diet can help to reduce the intake of harmful substances [50]. ROS presence has been studied in different animal models such as mice, rats, and fish.

The DCFH-DA fluorescence assay has been used to evaluate and quantify ROS production using in vitro and in vivo models, such as cell lines and fish, mice, and rat models. There are different methods to evaluate the inhibition of TBARS substances; one of these methods quantifies the oxidation of linoleic acid. Conjugated diene hydroperoxides with reactive AAPH can produce free radicals through molecular decomposition at 37 °C. These molecules produce radicals that react quickly with oxygen and causes lipid oxidation [51,52,53]. AAPH produce protein oxidation protein with increasing protein carbonyl content. AAPH is an effective reactor for the production of ROS in the in vitro and in vivo models. Exposure of proteins to either ^•^OH, O_2_^•−^, or both, leads to gross structural modifications that can produce protein fragmentation or cross-linking. Experimentally, alkylperoxyl radicals (RO_2_) can be produced by AAPH. The authors of other studies suggest that these free radicals can be responsible for protein oxidation [54,55,56]. In these processes, the inactivation of antioxidant enzymes (catalase, glutathione) has been described. These enzymes represent the important role of the antioxidant system in different organisms [57].

**Figure 5:**
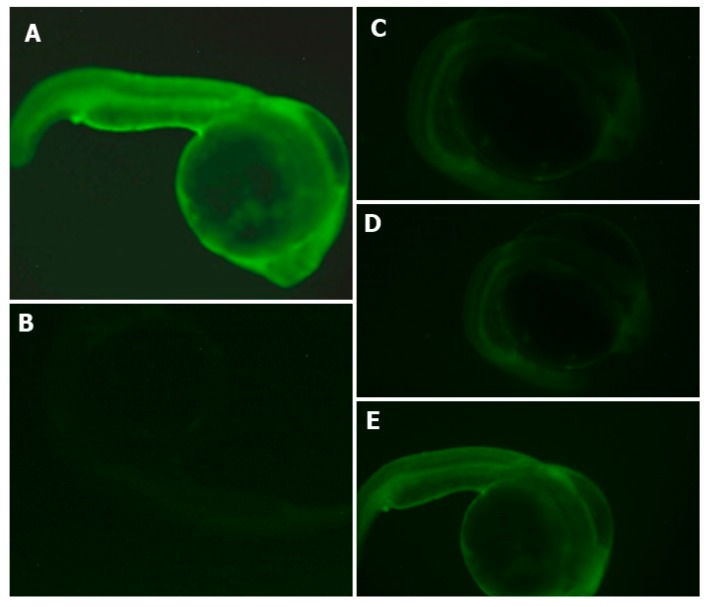
Effects of APC hydrolysates against AAPH-induced oxidative stress (ROS) in the zebrafish embryo model. (**A**) AAPH-treated group, (**B**) Non-treated group, (**C**) Gastric hydrolysate of APC at pH 2.0 and (**D**) intestinal hydrolysate of APC and (**E**) APC (amaranth protein concentrates without hydrolysis).

The percentage of survival of zebrafish embryos during the experiment was determined. The non-treated group presented a 100% survival rate of zebrafish embryos. The AAPH group presented a 75% survival rate, and the gastric and intestinal hydrolysates presented a value of 100% survival rate in zebrafish embryos. The antioxidant effects of APC hydrolysates (gastric and intestinal hydrolysate) against the intensity of ROS (DCFH-DA) is shown in Figure 5. The AAPH group presented a higher intensity of fluorescence. Gastric and duodenal APC hydrolysates were able to inhibit the formation of ROS in zebrafish larvae. The intensity of the fluorescence of both samples was lower than in the AAPH group. The APC sample presented a higher intensity of fluorescence. This is an indication that APC samples produce no protection in the production of ROS. The non-treated group presented the lowest intensity of fluorescence in all groups. It was difficult to obtain the microphotograph of the non-treated group, given that the intensity was extremely low.

**Figure 6:**
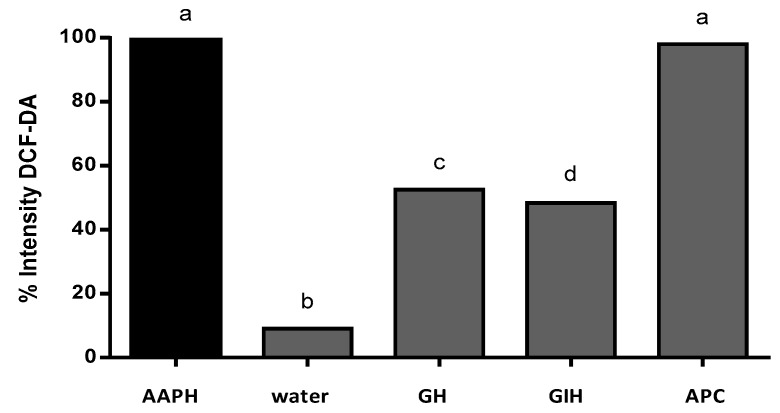
% of intensity by 2,7-dichlorofluorescein diacetate (DCFH-DA) fluorescence in the presence of AAPH. AAPH (positive control), water (basal control without AAPH), GH (gastric hydrolysate incubated with AAPH), IH (intestinal hydrolysate incubated with AAPH), and APC (amaranth protein concentrate incubated with AAPH). Results were analyzed using one-way ANOVA and followed by Tukey´s test. Different letters represents significant differences between samples as *p* < 0.05 (*n* = 3).

The intensity of the fluorescence was quantified using software to analyze the images (ImageJ). The intensity of fluorescence was compared to the positive control (AAPH). The result of the AAPH intensity fluorescence was classified as 100% intensity. Figure 6 shows the percentage of intensity of fluorescence of APC and its hydrolysates. Gastric hydrolysate at pH 2.0 presented 52.5% fluorescence intensity, and intestinal hydrolysates from APC presented 48.4% fluorescence intensity when compared to the AAPH signal. APC without hydrolysis presented 98% fluorescence intensity, this sample presented no protection against oxidation induced for AAPH. The non-treated group (control water) only presented 9.0% fluorescence intensity when compared to the AAPH group.

The use of AAPH as an inducer of ROS in the zebrafish model has been described in the literature. Kang et al. (2014) described ROS inhibition in zebrafish embryos with polysaccharide obtained from aloe vera plant (*Aloe barbadensis*) using DCFH-DA as a fluorescent chemical reactor [58]. Lee et al. reported the use of DCFH-DA for the analysis of ROS inhibition in zebrafish embryos treated with fucoidan obtained from *Ecklonia cava*; they reported the use of AAPH to produce oxidation in the zebrafish embryo model [59]. Other authors reported the use of DCFH-DA to inhibit ROS in cells. Goh et al. (2016), described ROS inhibition in keratinocyte cell lines, HaCaT, and monocytic THP-1 cell lines treated with concentrate obtained from *Alonia* species [60]. Jensen et al. (2015) reported ROS inhibition in polymorphonuclear cells in an extract obtained from blue-green algae [61].

It is known that oxygen-free radical substances can be used as a biological indicator in the evaluation of oxidative damage and oxidative stress [62,63,64,65].

## 4. Conclusions

APC proteins (*A. caudatus*) had showed a resistance to hydrolysis with pepsin during the gastric phase, while being susceptible to hydrolysis during the intestinal phase. APC protein hydrolysates produced in an in vitro gastrointestinal digestion model can inhibit lipid peroxidation under in vitro and in vivo conditions in the zebrafish larvae model. Fragments formed through hydrolysis were capable of inhibiting the production of ROS in the zebrafish embryos. The *A. caudatus* species is a promising source of antioxidant fragments that could exert a preventative effect against oxidative stress-associated diseases. Moreover, these fragments might be used as an additive to control the lipid oxidation responsible for the food deterioration during its manufacturing and storage. Further studies would be needed to identify, using LC-MS-MS, the bioactive peptides responsible for these observed effects, and to study the mechanisms of action for these fragments.
